# Two Nux Vomica Cases

**Published:** 1887-10

**Authors:** H. E. Potter

**Affiliations:** Clifton, Kan.


### TWO NUX VOMICA CASES.

H. E. Potter, M. D., Clifton, Kan.

A German (farmer), aged about thirty, came to me February 25th, 1886. The history of the case is as follows: About the first of the previous August he became unable to work because of dyspnœa on the slightest exertion; slight cough and indigestion were also present at this time. He began treatment with a regular (?) and continued, as he said, to take bottleful after bottleful of stuff till it would not stay in his stomach.

The doctors had first told him that he had "consumption," but after a time they concluded that *this* was complicated with "heart disease."

February 25th, when he came under my care, I could find no indications of structural change either of heart or lungs, though he was unable even to walk two blocks, because he couldn't "get his breath." On physical examination I found the temperature normal, skin moist and clear, heart action regular, full, and very rapid (one hundred and fifty per minute). The thorax was full, well-rounded, and the apices of lungs both full. Auscultation revealed nothing startling (slight subcrepitant sound). Respiration was quite rapid—thirty per minute—but the movements of the thorax were free and full.

Inquiring into his mode of living, I found that he was eating and sleeping well—too well, in fact—drinking from six to nine cups of strong coffee every day, and using an enormous quantity of tobacco, smoking and chewing almost constantly.

Subjective symptoms were very meagre, because of patient's inability to speak good English and mine to speak good German. The objective symptoms were not numerous either. But

I decided to give him Nux vomica, which I did, low, in form of No. 30 pellets.

I did not really expect to cure the case with one prescription, but it was all the medicine he ever took after coming into my hands, and during the year 1886 and up to the present time he has enjoyed perfect health, doing all the work incident to farm life. Of course, I insisted on a more rational diet and mode of living.

Case No. 2.—Mrs. B., æt. thirty-five, has been troubled with hæmorrhoids for years. Just recently passed through her second confinement. I was consulted about the seventh month of pregnancy, when the husband informed me that he was too poor to employ a doctor, and should therefore engage a midwife. I did not prescribe at this time, but recommended some dietary measures.

The lady finally had quite a tedious labor, with subsequent flooding, which was stopped by the midwife, or nature, when the patient was exhausted. Then the woman was left in her filth, "because it wouldn't be safe to use any water."

On the third day the husband again consulted me, stating that his wife had not slept for two nights (notwithstanding the fact that she had taken over a grain and a half of Morphia during this time) because of the terrible pain in her rectum. The "piles had *come down* during labor," been replaced by the patient, and again come down, with excruciating pain *all the time.*

There was no pain or soreness of abdomen or genital apparatus; it had all centered at the rectum. Now, I was not asked to prescribe, but simply consulted as to the advisability of giving oil, as the midwife was about to perform this bit of barbarity and the husband had some misgivings in the matter, fearing it *might* make the "piles" worse.

Of course, I told him to avoid oil as he would the devil, gave him some Nux, and told him how to administer it. The remedy was given in this case because I knew of no other with the symptom "all pains concentrate at the rectum." The result was what a homœopath would expect, viz.: immediate relief, a good night's sleep, and improvement generally.

I wanted to give this second case China, but on considering the fact that the trouble was *not brought on* by "loss of blood or other vital fluid," but had existed long before this loss occurred, other symptoms being so meagre, and *the one* calling for Nux standing out so clearly, led me to *the remedy.*

In the first instance I gave Nux to clear up the case after the fellow had been drugged, and found it was a Nux case all the time.

